# Health Equity of Hypertension Management Between Local Residents and Internal Migrants in Shenzhen, China: Cross-Sectional Study

**DOI:** 10.2196/65548

**Published:** 2025-02-10

**Authors:** Jinyu He, Yanjun Li, Huatang Zeng, Haoran Sun, Liqun Wu, Zhenzhen Zhu, Ning Zhang, Wannian Liang

**Affiliations:** 1 Vanke School of Public Health, Tsinghua University, Beijing, China; 2 Institute of Healthy China, Tsinghua University, Haidian District, Beijing, 100084, China, 86 13772418478, 86 010 62799645; 3 Public Hygiene and Health Commission of Shenzhen Municipality, Shenzhen Health Development Research and Data Management Center, Shenzhen, China

**Keywords:** health equity, hypertension management, immigrant population, hypertension, China, global health, public health, health disparities, medical record, community health care, native population, immigrant, socioeconomic, disease burden, well-being, chronic disease, community health center

## Abstract

**Background:**

With hypertension emerging as a global public health concern, taking measures to alleviate its burden is urgently needed. The hypertension management program (HMP) in China is a standardized policy to help people with hypertension to improve their health levels and reduce health inequalities. However, studies focusing on details regarding participation in this program remain scarce.

**Objective:**

This study aims to investigate the participation rates in HMPs in China and examine the health disparities in hypertension management between local residents and internal migrants in Shenzhen.

**Methods:**

This study used the medical record of Shenzhen, Guangdong, China. We included adults with new-onset diagnosis of hypertension after 2017 and focused on patients who have a record in the community health center. We described the basic characteristics of people diagnosed with hypertension, including age, gender, marital status, occupation, education level, and health insurance type. Enrolled rate, follow-up rate, and adherence rate were used to measure the engagement with this program at the city, district, and community levels.

**Results:**

Of the 1,160,214 hypertensive patients, 29.70% (344,526/1,160,214) were local residents, while 70.3% (815,688/1,160,214) were internal migrants. In addition, 55.36% (642,250/1,160,214) were enrolled in the HMP. Of those, 57.52% (369,439/642,25) were followed up. In addition, 62.59% (231,217/369,439) of followed up individuals were adherents. Internal migrants demonstrated a significantly higher participation in the HMP, especially for the enrolled rate (local residents: 156,085/344,526, 45.30% vs internal migrants: 486,165/815,688, 59.60%) and adherence rate (local residents: 44,044/84,635, 52.04% vs internal migrants: 187,173/284,804, 65.72%). Apart from that, local, single, and younger individuals had lower rates compared to their counterparts. There also existed within-district and within-community variation among enrolled, follow-up, and adherence rates.

**Conclusions:**

Based on our research, individuals with different demographic and socioeconomic characteristics and in different regions had different enrolled, follow-up, and adherence rates. Internal migrants in Shenzhen showed a significantly higher participation in the HMP. Given these findings, there exists the potential to enhance the outreach and engagement of local, single, and younger populations through targeted promotional strategies.

## Introduction

### Background

The United Nations’ Sustainable Development Goals are important global issues. The goals focus on promoting health and well-being, as well as reducing inequalities, highlighting the need for all individuals, regardless of their circumstances, to be able to access quality health care and essential services for optimal health outcomes.

Hypertension presents a significant global health challenge, emerging as a primary contributor to the global disease burden. Analysis from the Global Burden of Disease Study reveals that high systolic blood pressure stands as the foremost risk factor for mortality worldwide, accounting for 10.8 million cardiovascular disease deaths and 11.3 million deaths overall in 2021 [1]. The World Health Organization’s latest report highlights a surge in hypertension prevalence, with figures doubling from 650 million individuals in 1990 to an alarming 1.3 billion in 2019 [2]. In China, about 270 million people have hypertension, while research showed that, among the 256 million individuals aged 30-79 years with hypertension, only 52% were aware of their condition, 39% were receiving treatment, and a scant 16% had their blood pressure under control [2,3]. In China, hypertension is one of the earliest chronic diseases undergoing widespread public health intervention. The government has bolstered efforts to combat hypertension through initiatives such as the Basic Public Health Services policy, China Medium- and Long-term Plan for the Prevention and Treatment of Chronic Diseases, National Demonstration Areas for Comprehensive Chronic Disease Prevention and Control, and the National Healthy Lifestyle Action. Chinese strategies underscore the inclusion of hypertension management within basic public health services at the grassroots level, ensuring regular dissemination of national guidelines on hypertension management and treatment. These guidelines provide comprehensive details on the management processes and protocols for diagnosed patients and vulnerable individuals [4]. According to the National Standard for Basic Public Health Services [5], the hypertension management program (HMP) is one of most crucial programs within the Basic Public Health Services. It requires community health centers (CHCs) to establish dedicated medical records for hypertensive patients and provide regular follow-up services without additional fees beyond outpatient costs. According to performance assessments, there must be at least 1 follow-up visit record every 3 months.

Significant health inequities exist in the health policy context; health-related behaviors and beliefs; quality of care; utilization of health care services; and health status [6]. Research has highlighted health inequities between local residents and internal migrants in China. Many internal migrants still face significant barriers in accessing local public health services, particularly in establishing health records and receiving health education. This is more common in economically developed eastern regions, where internal migrants use public health services less often than those in central regions, indicating regional disparities [7]. Furthermore, internal migrants often have poorer health compared to local residents due to socioeconomic and cultural factors, with social integration and income inequality being key determinants. In 2017, internal migrants in Qingdao had relatively better health, while those in Shenzhen faced the greatest health challenges [8].

In terms of hypertension management, treatment, and control within the population [9], previous research has explored the potential impacts of multidimensional factors on the health inequity of hypertension and cardiovascular disease [10]; the factors can be summarized as individual, socioeconomic [11,12], and environmental. A nationally representative cross-sectional study in China revealed that age, BMI, and economic status contributed to the inequitable situation of hypertension prevalence [13]. Another study showed that economic status and educational level are associated with socioeconomic inequalities of health services utilization among patients with hypertension in the Pearl River Delta of China [14]. In addition, due to economic status, the hypertension burden appeared higher in rural areas compared to urban regions in China [15].

As China’s first Special Economic Zone, Shenzhen has undergone sweeping changes, including a significant demographic shift due to a large influx of internal migrants from other parts of Mainland China. According to The Shenzhen Statistical Yearbook 2022, the total population of Shenzhen was 17,681,600, of which the registered household population was 5,563,900 (31.5%) and the nonregistered population was 12,117,700 (68.5%). Despite this, Shenzhen, similar to many other regions in China, prioritizes the provision of health care services to its local residents [16]. For example, older adults with local household registrations or local medical insurance are prioritized for free influenza vaccination, while internal migrants without local insurance have to pay out of pocket [17]. Local people aged ≥60 years are eligible for free health checks, whereas internal migrants are required to be ≥65 years old [18].

As a survey indicated that the prevalence rate of hypertension among the residents of Shenzhen is 20.74% [5], and the incidence of hypertension in Shenzhen is increasing, the HMP is accessible to all patients regardless of household registration status, with the aim of reducing health disparities; however, as of yet, there has been no research comparing the health inequity in hypertension management between internal migrants and local residents. In the context of an internal migrant–dense city, understanding the unique challenges and needs of both local residents and internal migrants is essential.

### This Study

The primary objective of this baseline report was to describe the characteristics of hypertensive patients and those who have received HMP services at a CHC, as well as the current status of the specific services they receive. The secondary objective was to reveal the health inequity for utilization of hypertension management between local residents and internal migrants in Shenzhen.

## Methods

### Study Design and Data Source

This study used a cross-sectional design. The data of the study were obtained from CHCs and the government-owned hospitals in Shenzhen, Guangdong, China. All data were extracted with SQL queries from original datasets at the Shenzhen Health Development Research and Data Management Center. The data included the patients’ basic information as well as the actual hypertension record and follow-up service record from January 1, 2017, to September 30, 2023. We defined participants without Shenzhen household registration who had been living in Shenzhen for 6 months or more as internal migrants. The HMP is a key component of primary care services for hypertensive patients. Therefore, this study uses the enrollment rate, follow-up rate, and adherence rate of this program to assess the health equity in utilization of primary care services between local residents and internal migrants.

### Eligibility Criteria of Resident Records

We included the records of the following residents: (1) those who were documented in either the health information system of a CHC or medical record of government-owned hospitals in Shenzhen, Guangdong, China from January 1, 2017, to September 30 2023; (2) those who were diagnosed with hypertension according to these information systems (patients with gestational hypertension, based on the International Classification of Diseases Tenth Revision code, were excluded); (3) those whose earliest diagnosis date was after January 1, 2017, and (4) those who were aged ≥18 years, which is the age threshold for blood pressure measurements when people visit medical institutions as per the hypertension management policy in Shenzhen.

### Sociodemographic Variables

A total of 6 individual-level sociodemographic factors were included in this study: gender (male or female), age (<45 years, 45‐65 years, and >65 years), marital status (single, married, widowed, or divorced), occupation (technical personnel/office staff, business service industry, agriculture and forestry, industry, other, unemployed), education level (Bachelor’s degree or above, college diploma, high school or technical school, junior high school, elementary school or below), and health insurance type (urban employees, urban residents, others, fully self-funded).

### Key Variables

The participation in the HMP in this research was assessed using 3 measures. First, “enrollment” in the program was defined as having a hypertensive record, which includes the diagnosis date, blood pressure at the time of diagnosis, lifestyle, specific information about the physicians and institutions providing the services, and patient contact information. This indicates that the patient has visited the CHC and the doctor had the opportunity to interact with them. Enrollment in the HMP is the initial step for doctors when encountering a hypertensive patient for the first time according to the policy. Second, follow-up in this study was defined as patients having at least 2 follow-up records at different times. Third, regular follow-up was defined as patients having an average of more than 4 follow-up visits per year, in accordance with the national policy, which mandates that hypertensive patients should be followed up at least 4 times per year. We used 3 corresponding ratios to evaluate engagement. The enrolled rate refers to the proportion of individuals enrolled out of all patients who have ever been diagnosed with hypertension. The follow-up rate indicates the number of individuals who are followed up out of those enrolled. The adherence rate represents the proportion of individuals who undergo regular follow-up out of the total number of those who are followed up.

### Statistical Analysis

Individuals were assessed by household register (internal migrants or local residents). Indicators were estimated for participation in the HMP; for the whole city, this was stratified by sociodemographic factors (resident status, gender, age category, occupation, education level, and insurance type). The city was also stratified by district and community. Categorical variables and prevalence rates were expressed as absolute frequencies and percentages. Missing values were categorized as 1 group for each variable. Logistic regression was used to evaluate the main characteristics associated with participation in the HMP among all patients and internal migrants and local residents as a sensitivity analysis. A 2-sample *z*-test for proportions was used to evaluate whether the observed differences in proportions between internal migrants and local residents were statistically significant. The map figure was drawn using ArcMap 10.8 (Esri) and the other figures were drawn using OriginPro (version 2020b; OriginLab). Statistical analyses were conducted using R Studio (version 4.2.2; Posit).

### Ethical Considerations

The data used in this study were anonymized and encrypted, ensuring no access to personally identifiable information. Our data access was authorized through an agreement with government departments. In addition, our study is 1 component of a larger study that was approved by The Tsinghua University Science and Technology Ethics Committee (20230065).

## Results

### Demographic Characteristics

The total number of diagnosed individuals recorded in the electronic health record was 1,160,214, with 344,526 local residents (29.7%) and 815,688 internal migrants (70.3%). In the overall population, 58.24% (n=675,692) were male and 41.76% (n=484,522) were female. More demographic characteristics of the diagnosed population are detailed in Table 1. Males constituted a higher proportion across all age groups and resident categories, with a noticeable decline in older age groups. Higher educational attainment was more prevalent in younger age groups. Internal migrants had higher proportions of individuals with lower educational levels (elementary school or below). Insurance coverage varied, with urban employee insurance being more common in younger and middle-aged groups, while urban resident insurance was more prevalent in the oldest age group.

**Table 1.** Characteristics of residents with hypertension from the electronic health record in Shenzhen by age group (January 1, 2017, to September 30, 2023).

	Total (n=1,160,214), n (%)				Local population (n=344,526), n (%)				Migrant population (n=815,688), n (%)			
Characteristics	18‐45 years (n=279,179)	46‐65 years (n=609,238）	>65 years (n=271,797）	Subtotal	18‐45 years (n=72,361)	46‐65 years (n=175,887)	>65 years (n=96,278)	Subtotal	18‐45 years (n=206,818)	46‐65 years (n=433,351)	>65 years (n=175,519)	Subtotal
Sex												
Male	195,225 (69.9)	356,461 (58.5)	124,006 (45.6)	675,692 (58.2)	47,125 (65.1)	102,826 (58.5)	46,050 (47.8)	196,001 (56.9)	148,100 (71.6)	253,635 (58.5)	77,956 (44.4)	479,691 (58.8)
Female	83,954 (30.1)	252,777 (41.5)	147,791 (54.4)	484,522 (41.8)	25,236 (34.9)	73,061 (41.5)	50,228 (52.2)	148,525 (43.1)	587,18 (28.4)	179,716 (41.5)	97,563 (55.6)	335,997 (41.2)
Marital status												
Single	62,445 (22.4)	35,202 (5.8)	11,072 (4.1)	108,719 (9.4)	18,165 (25.1)	16,762 (9.5)	7141 (7.4)	42,068 (12.2)	44280 (21.4)	18,440 (4.3)	3931 (2.2)	66,651 (8.2)
Married	213,769 (76.6)	567,735 (93.2)	249,868 (91.9)	1,031,372 (88.9)	53,405 (73.8)	156,995 (89.3)	84,909 (88.2)	295,309 (85.7)	160,364 (77.5)	410,740 (94.8)	164,959 (94.0)	736,063 (90.2)
Widowed or divorced	1860 (0.7)	4738 (0.8)	10,293 (3.8)	16,891 (1.5)	358 (0.5)	1553 (0.9)	3935 (4.1)	5846 (1.7)	1502 (0.7)	3185 (0.7)	6358 (3.6)	11,045 (1.4)
Missing	1105 (0.4)	1563 (0.3)	564 (0.2)	3,232 (0.3)	433 (0.6)	577( 0.3)	293 (0.3)	1303 (0.4)	672 (0.3)	986 (0.2)	271 (0.2)	1929 (0.2)
Occupation												
Technical personnel or office staff	58,423 (20.9)	86,191 (14.1)	34,917 (12.8)	179,531 (15.5)	25,601 (35.4)	49,862 (28.3)	21,889 (22.7)	97,352 (28.3)	32,822 (15.9)	36,329 (8.4)	13,028 (7.4)	82,179 (10.1)
Business service industry	68,022 (24.4)	134,895 (22.1)	20,419 (7.5)	223,336 (19.2)	18,089 (25.0)	41,752 (23.7)	11,077 (11.5)	70,918 (20.6)	49,933 (24.1)	93,143 (21.5)	9342 (5.3)	152,418 (18.7)
Agriculture and forestry	2617 (0.9)	13,573 (2.2)	23,599 (8.7)	39,789 (3.4)	432 (0.6)	2035 (1.2)	4213 (4.4)	6680 (1.9)	2185 (1.1)	11,538 (2.7)	19,386 (11.0)	33,109 (4.1)
Industry	70,811 (25.4)	114,431 (18.8)	9548 (3.5)	194,790 (16.8)	3024 (4.2)	6877 (3.9)	3988 (4.1)	13,889 (4.0)	67,787 (32.8)	107,554 (24.8)	5560 (3.2)	180,901 (22.2)
Other	37,624 (13.5)	78,876 (12.9)	26,787 (9.9)	143,287 (12.4)	16,709 (23.1)	38,393 (21.8)	16,096 (16.7)	71,198 (20.7)	20,915 (10.1)	40,483 (9.3)	10,691 (6.1)	72,089 (8.8)
Unemployed	41,271 (14.8)	180,768 (29.7)	156,430 (57.6)	378,469 (32.6)	8463 (11.7)	36,932 (21.0)	38,987 (40.5)	84,382 (24.5)	32,808 (15.9)	143,836 (33.2)	117,443 (66.9)	294,087 (36.1)
Missing	411 (0.1)	504 (0.1)	97 (0.0)	1012 (0.1)	43 (0.1)	36 (0.0)	28 (0.0)	107 (0.0)	368 (0.2)	468 (0.1)	69 (0.0)	905 (0.1)
Education level												
Bachelor’s degree or above	42,576 (15.3)	43,995 (7.2)	10,930 (4.0)	97,501 (8.4)	25,497 (35.2)	33,534 (19.1)	7483 (7.8)	66,514 (19.3)	17,079 (8.3)	10,461 (2.4)	3447 (2.0)	30,987 (3.8)
College diploma	46,343 (16.6)	51,669 (8.5)	15,667 (5.8)	113,679 (9.8)	17,230 (23.8)	31,359 (17.8)	9634 (10.0)	58,223 (16.9)	29,113 (14.1)	20,310 (4.7)	6033 (3.4)	55,456 (6.8)
High school or technical school	84,781 (30.4)	164,361 (27.0)	52,107 (19.2)	301,249 (26.0)	13,239 (18.3)	53,872 (30.6)	24,805 (25.8)	91,916 (26.7)	71,542 (34.6)	110,489 (25.5)	27,302 (15.6)	209,333 (25.7)
Junior high school	64,310 (23.0)	194,959 (32.0)	65,548 (24.1)	324,817 (28.0)	3196 (4.4)	23,497 (13.4)	20,121 (20.9)	46,814 (13.6)	61,114 (29.5)	171,462 (39.6)	45,427 (25.9)	278,003 (34.1)
Elementary school or below	5205 (1.9)	91,713 (15.1)	108,233 (39.8)	205,151 (17.7)	372 (0.5)	7699 (4.4)	22,928 (23.8)	30,999 (9.0)	4833 (2.3)	84014 (19.4)	85,305 (48.6)	174,152 (21.4)
Missing	35,964 (12.9)	62,541 (10.3)	19,312 (7.1)	117,817 (10.2)	12,827 (17.7)	25,926 (14.7)	11,307 (11.7)	50,060 (14.5)	23,137 (11.2)	36,615 (8.4)	8005 (4.6)	67,757 (8.3)
Insurance type												
Urban employees	167,374 (60.0)	277,068 (45.5)	33,538 (12.3)	477,980 (41.2)	43,049 (59.5)	96,562 (54.9)	27,879 (29.0)	167,490 (48.6)	124,325 (60.1)	180,506 (41.7)	5659 (3.2)	310,490 (38.1)
Urban residents	27,268 (9.8)	74,175 (12.2)	40,742 (15.0)	142,185 (12.3)	11,510 (15.9)	44,050 (25.0)	35,570 (36.9)	91,130 (26.5)	15,758 (7.6)	30,125 (7.0)	5172 (2.9)	51,055 (6.3)
Other	65,806 (23.6)	222,946 (36.6)	184,410 (67.8)	473,162 (40.8)	8336 (11.5)	17,126 (9.7)	24,302 (25.2)	49,764 (14.4)	57,470 (27.8)	205,820 (47.5)	160,108 (91,.2)	423,398 (51.9)
Fully self-funded	1085 (0.4)	2681 (0.4)	1406 (0.5)	5172 (0.4)	458 (0.6)	749 (0.4)	308 (0.3)	1515 (0.4)	627 (0.3)	1932 (0.4)	1098 (0.6)	3657 (0.5)
Missing	17,646 (6.3)	32,368 (5.3)	11,701 (4.3)	61,715 (5.3)	9008 (12.4)	17,400 (9.9)	8219 (8.5)	34,627 (10.1)	8638 (4.2)	14,968 (3.5)	3482 (2.0)	27,088 (3.3)

### City-Level HMP Participants

#### In Total

**Figure 1.**
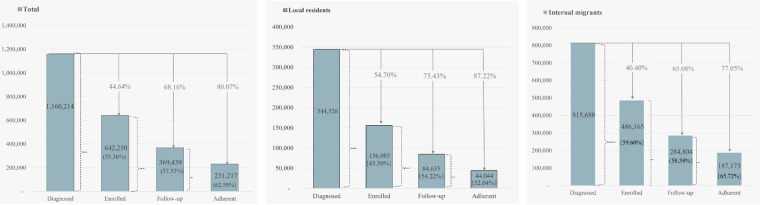
City-level hypertension management program participants by household registration.

Among all diagnosed individuals, 55.36% (n=642,250) were enrolled in the HMP. We found that 57.52% (n=369,429) of those followed up. Furthermore, 62.59% (n=231,217) of the individuals who were followed up demonstrated adherence. Compared to local residents, internal migrants have a significantly higher participation rate (*P*<.001) in the HMP, as shown in Figure 1 (Table S1 in Multimedia Appendix 1).

#### Enrollment Rate

Among all diagnosed individuals (n=1,160,214), 58.1% (n=642,250) were enrolled in the HMP. As is shown in Table 2, the enrollment rates were similar between different sexes, with males at 55.27% (n=373,452) and females at 55.48% (n=268,798). For internal residents, the enrollment rate was higher for females, with a rate of 60.15% (202,115/335,997) for females and 59.22% (284,050/479,691) for males. The opposite was seen for local migrants. Industrial workers had the highest enrollment rate, followed by those in agriculture and forestry, then the unemployed, with the lowest rate among those in the “other” category, and technical and administrative personnel just above that. For the local population, the business service industry was above the “other” group. The lower the educational level, the higher the enrollment rate. Older individuals had higher enrollment rates; the enrollment rate was only 39.27% (n=109,623) among those aged 18‐44 years, but it increased to 64.14% (n=174,335) among those older than 65 years. Divorced and widowed individuals represented the smallest proportion of the diagnosed population, but they had the highest enrollment rate at 67.52% (n=11,405), followed by married individuals at 59.16% (n=610,208), with single individuals having the lowest rate at 18.41% (n=20,018). The trends in enrolled rates across different age groups, marital statuses, and educational levels were similar between local residents and internal migrants. The logistic regression results indicated that all the research variables examined were significant factors influencing the enrollment rate (Table S2 in Multimedia Appendix 2).

**Table 2.** Participation rates in hypertension management programs by household registration in Shenzhen (January 1, 2017, to September 30, 2023).

Indicator	Enrolled individuals, n (%)^a^			Follow-up individuals, n (%)^a^			Adherent individuals, n (%)^a^		
Characteristics	Total (n=642,250)	Local residents (n=156,085)	Internal migrants (n=486,165)	Total (n=369,439)	Local residents (n=84,635)	Internal migrants (n=284,804)	Total (n=231,217)	Local residents (n=44,044)	Internal migrants (n=187,173)
Gender									
Male	373,452 (55.27)	89,402 (45.61)	284,050 (59.22)	220,066 (58.93)	48,848 (54.64)	171,218 (60.28)	140,515 (63.85)	26,207 (53.65)	114,308 (66.76)
Female	268,798 (55.48)	66,683 (44.9)	202,115 (60.15)	149,373 (55.57)	35,787 (53.67)	113,586 (56.2)	90,702 (60.72)	17,837 (49.84)	72,865 (64.15)
Age group (years)									
18‐44	109,623 (39.27)	18,782 (25.96)	90,841 (43.92)	65,685 (59.92)	9565 (50.93)	56,120 (61.78)	45,436 (69.17)	5950 (62.21)	39,486 (70.36)
45‐64	358,292 (58.81)	83,580 (47.52)	274,712 (63.39)	206,746 (57.7)	45,131 (54)	161,615 (58.83)	127,871 (61.85)	23,454 (51.97)	104,417 (64.61)
≥65	174,335 (64.14)	53,723 (55.8)	120,612 (68.72)	97,008 (55.64)	29,939 (55.73)	67,069 (55.61)	57,910 (59.7)	14,640 (48.9)	43,270 (64.52)
Marital status									
Single	20,018 (18.41)	5240 (12.46)	14,778 (22.17)	10,057 (50.24)	2428 (46.34)	7629 (51.62)	7166 (71.25)	1451 (59.76)	5715 (74.91)
Married	610,208 (59.16)	147,202 (49.85)	463,006 (62.90)	352,829 (57.82)	80,168 (54.46)	272,661 (58.89)	220,041 (62.36)	41,532 (51.81)	178,509 (65.47)
Widowed or divorced	11,405 (67.52)	3412 (58.36)	7993 (72.37)	6426 (56.34)	2001 (58.65)	4425 (55.36)	3923 (61.05)	1039 (51.92)	2884 (65.18)
Missing	619 (19.15)	231 (17.73)	388 (20.11)	127 (20.52)	38 (16.45)	89 (22.94)	87 (68.5)	22 (57.89)	65 (73.03)
Occupation									
Technical personnel or office staff	91,870 (51.17)	47,737 (49.04)	44,133 (53.7)	52,918 (57.6)	26,918 (56.39)	26,000 (58.91)	29,624 (55.98)	13,365 (49.65)	16,259 (62.53)
Business service industry	122,120 (54.68)	34,758 (49.01)	87,362 (57.32)	70,627 (57.83)	18,956 (54.54)	51,671 (59.15)	43,445 (61.51)	9998 (52.74)	33,447 (64.73)
Agriculture and forestry	24,092 (60.55)	3577 (53.55)	20,515 (61.96)	13,895 (57.67)	2015 (56.33)	11,880 (57.91)	8382 (60.32)	1043 (51.76)	7339 (61.78)
Industry	134,668 (69.13)	7855 (56.56)	126,813 (70.1)	88,760 (65.91)	4568 (58.15)	84,192 (66.39)	58,049 (65.4)	2405 (52.65)	55,644 (66.09)
Other	44,554 (31.09)	18,754 (26.34)	25,800 (35.79)	21,457 (48.16)	9216 (49.14)	12,241 (47.45)	12,915 (60.19)	4779 (51.86)	8136 (66.47)
Unemployed	224,840 (59.41)	43,397 (51.43)	181,443 (61.7)	121,778 (54.16)	22,962 (52.91)	98,816 (54.46)	78,798 (64.71)	12,454 (54.24)	66,344 (67.14)
Missing	106 (10.47)	7 (6.54)	99 (10.94)	4 (3.77)	0 (0)	4 (4.04)	4 (100)	0 (0)	4 (100)
Education level									
Bachelor’s degree or above	40,803 (41.85)	28,613 (43.02)	12,190 (39.34)	21,729 (53.25)	15,248 (53.29)	6481 (53.17)	11,926 (54.89)	7798 (51.14)	4128 (63.69)
College diploma	53,614 (47.16)	27,111 (46.56)	26,503 (47.79)	29,547 (55.11)	14,640 (54)	14,907 (56.25)	17,369 (58.78)	7610 (51.98)	9759 (65.47)
High school or technical school	169,218 (56.17)	47,939 (52.16)	121,279 (57.94)	98,505 (58.21)	26,429 (55.13)	72,076 (59.43)	59,936 (60.85)	13,514 (51.13)	46,422 (64.41)
Junior high school	212,807 (65.52)	26,355 (56.3)	186,452 (67.07)	127,916 (60.11)	14,595 (55.38)	113,321 (60.78)	82,575 (64.55)	7776 (53.28)	74,799 (66.01)
Elementary school or below	144,562 (70.47)	18,481 (59.62)	126,081 (72.4)	83,165 (57.53)	10,490 (56.76)	72,675 (57.64)	54,244 (65.22)	5547 (52.88)	48,697 (67.01)
Missing	21,246 (18.03)	7586 (15.15)	13,660 (20.16)	8577 (40.37)	3233 (42.62)	5344 (39.12)	5167 (60.24)	1799 (55.64)	3368 (63.02)
Insurance type									
Urban employees	270,312 (56.55)	80,523 (48.08)	189,789 (61.13)	166,048 (61.43)	44,582 (55.37)	121,466 (64)	99,579 (59.97)	22,840 (51.23)	76,739 (63.18)
Urban residents	81,890 (57.59)	51,233 (56.22)	30,657 (60.05)	46,235 (56.46)	28,688 (56)	17,547 (57.24)	25,561 (55.28)	14,726 (51.33)	10,835 (61.75)
Fully self-funded	280,172 (59.21)	20,301 (40.79)	259,871 (61.38)	153,701 (54.86)	9797 (48.26)	143,904 (55.38)	103,928 (67.62)	5586 (57.02)	98,342 (68.34)
Other	2519 (48.70)	456 (30.1)	2063 (56.41)	1352 (53.67)	217 (47.59)	1135 (55.02)	878 (64.94)	130 (59.91)	748 (65.9)
Missing	7357 (11.92)	3572 (10.32)	3785 (13.97)	2103 (28.59)	1351 (37.82)	752 (19.87)	1271 (60.44)	762 (56.4)	509 (67.69)

a All percentages are calculated in relation to a broader population. For instance, the proportion of males within the enrolled individuals stands at 55.27% and is calculated as n/N × 100. Here, "n" represents the number of males within the enrolled individuals subset, while "N" denotes the number of males within the entire population. For N values, please refer to the corresponding "subtotal" columns in Table 1.

#### Follow-Up Rate

Of patients who have been enrolled in the hypertension program, about 57.52% (369,439/642,250) were followed up. As is shown in Table 2, the follow-up rate was higher among internal migrants (284,804/486,165, 58.58%), while it was 54.22% (84,635/156,085) among the local population. The proportions of those who follow up were higher among men (male: 220,066/373,452, 58.93%; female: 149,373/268,798, 55.57%) and industrial workers (88,760/134,668, 65.91%). For other characteristics, there were significant differences in follow-up rates between local residents and internal migrants. For the local population, follow-up rates increased with age: 50.93% (9565/18,782) for those aged 18‐44 years and 55.73% (29,939/53,723) for those over 65 years. However, for internal migrants, the trend was the opposite: the follow-up rate for those aged 18‐44 years was 61.78% (56,120/90,841), while it was 55.61% (67,069/120,612) for those over 65 years. Among local residents, the highest follow-up rate was observed among divorced and widowed individuals, at 58.65% (2001/3412). Conversely, for internal migrants, married individuals had the highest follow-up rate, at 58.89% (272,661/463,006). Single individuals exhibited the lowest follow-up rates in both populations, with 46.34% (2428/5240) of local residents and 51.62% (7629/14,778) of internal migrants following up. Among local residents, the highest follow-up rate was observed in individuals with an educational level of elementary school or below and those with urban resident insurance. Conversely, among internal migrants, the highest follow-up rate was found in individuals with a junior high school education and those with urban employee insurance. The logistic regression results indicated that all the research variables examined were significant factors influencing the follow-up rate (Table S2 in Multimedia Appendix 2).

#### Adherence Rate

A total of 62.59% (231,217/369,439) of patients who followed up continued to do so at least 4 times per year. As is shown in Table 2, similar to the enrolled rate and follow-up rate, the adherence rate was higher among internal migrants (187,173/284,804, 65.72%) compared to local residents (44,044/84,635, 52.04%). Among the followed-up patients, men (140,515/220,066, 63.85%), single individuals (7166/10,057, 71.25%), and people aged 18‐44 years (45,436/65,685, 69.17%) had the highest adherence rates. Individuals with urban employee and urban resident insurance did not have the highest adherence rates. These trends were consistent between both local and internal migrants. However, among local residents, those engaged in commercial and industrial occupations had the highest adherence rates, at 52.74% (9998/18,956) and 52.65% (2405/4568), respectively. Conversely, in the internal migrants group, the unemployed and those classified under other categories had the highest adherence rates, at 67.14% (66,344/98,816) and 66.47% (8136/12,241), respectively. Regarding educational level, among local residents, those with a junior high school level of education had the highest adherence rate at 53.28% (7776/14,595), whereas among internal migrants, those with an educational level of elementary school or below had the highest adherence rate. The logistic regression results indicated that all the research variables examined were significant factors influencing the adherence rate (Table S2 in Multimedia Appendix 2).

### District-Level Hypertension Management

**Figure 2.**
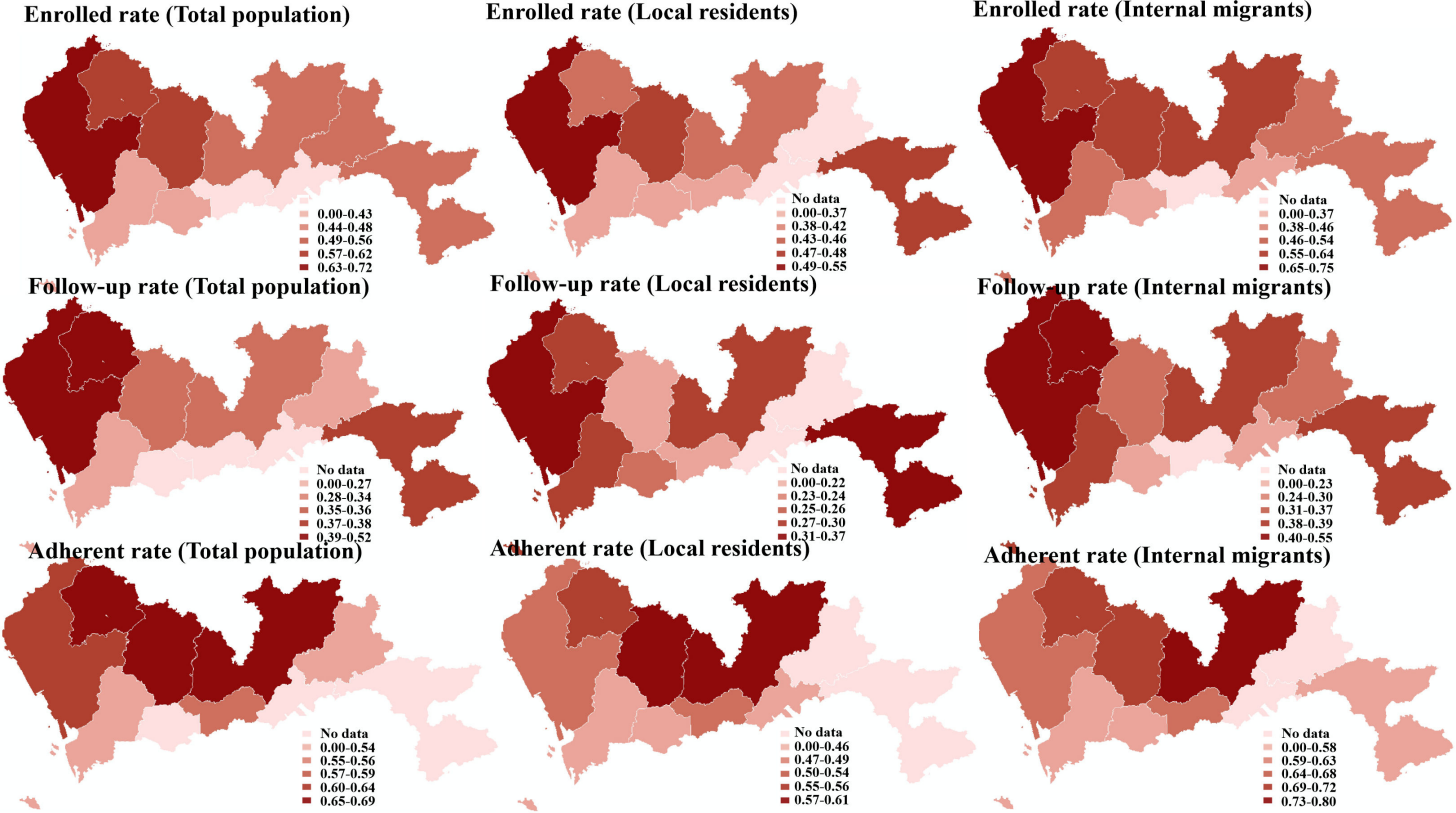
District-level hypertension management.

As shown in Figure 2, the highest enrolled rate was detected in Baoan district (180,301/250,036, 72.11%), Guangming district (33,308/54,063, 61.61%), and Longhua district (74,115/124,878, 59.35%), while the lowest enrolled rate was found in Luohu district (52,413/132,023, 39.70%), Yantian district (8609/19,924, 43.21%), and Futian district (59,962/132,337, 45.31%). It is worth noting that, apart from Luohu district, the enrolled rate of internal migrants was higher than that of local residents.

Regarding follow up rate, Baoan district, Guangming district, and Dapeng New Area showed the highest rates, which were 52.21% (131,171/251,237), 45.86% (24,915/54,328), and 36.72% (4301/11,713), while the lowest rate was found in the same places as the enrolled rate. The enrolled rate of internal migrants was higher than that of local residents in all districts.

Considering the adherence rate, Longhua district (31,779/45,917, 69.21%), Longgang district (54,088/79,965, 67.64%), and Guangming district (17,949/26,658, 67.33%) were ranked as the first 3. Similarly, the adherence rate of internal migrants was higher than that of local residents in all districts.

### Community-Level Hypertension Management

**Figure 3.**
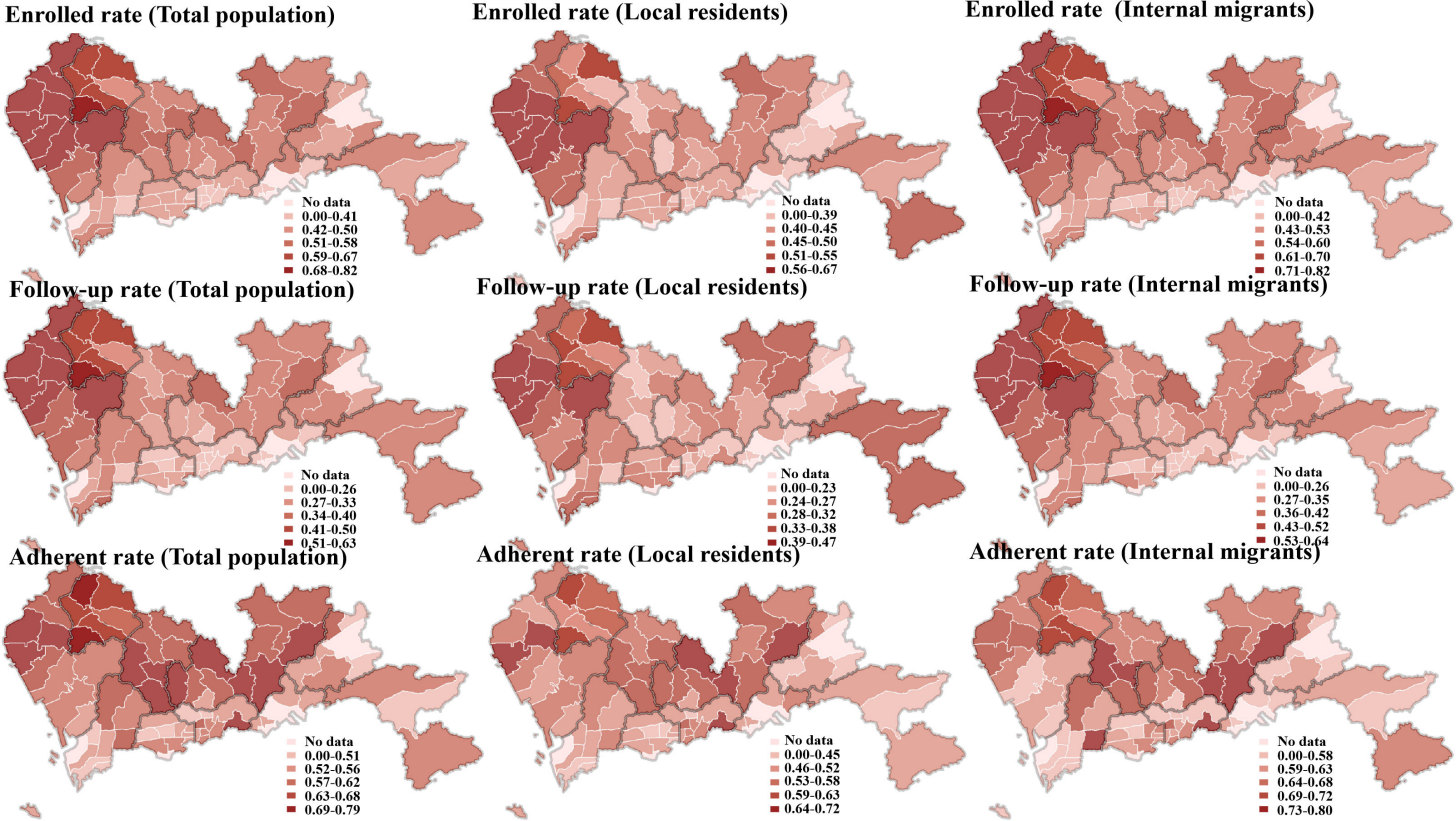
Community-level hypertension management.

There was considerable within-city and within-district variation in the community level of hypertension management. Generally speaking, the community-level enrolled rate, follow-up rate, and adherence rate were descending from east to west and from north to south. Although only the adherence rate was slightly different, some communities in the southern region showed a high rate, as shown in Figure 3.

There also exists within-district variation such that a 24.30% gap between different communities was found in the enrollment rate of Guangming district. A 23.59% gap was detected in the follow-up rate of Baoan district. Similarly, a 23.59% gap was found in the adherence rate in Luohu district. In addition, rates of internal migrants were much higher than those of local residents in all districts.

## Discussion

### Principal Results

Regarding the crucial role of health service supply from primary health centers in achieving health equity in the domain of hypertension prevention, we summarized the enrolled rate, follow-up rate, and adherence rate of the Shenzhen population that participated in the HMP based on the data collected from CHCs. Among all diagnosed individuals, 55.36% (642,250/1,160,214) were enrolled in the HMP. Of those, 57.52% (369,439/642,250) followed up, and 62.59% (231,217/369,439) were adherents. Furthermore, we compared the health inequity in hypertension between internal migrants and local residents in Shenzhen.

### Comparison With Prior Work

Overall, the data revealed that men exhibited higher rates of both follow-up and adherence compared to women, which is not consistent with the conclusion of some studies [19,20] related to hypertension treatment adherence. However, the medication adherence rates of men and women taking one medication were 61.04% and 55.86% in Beijing, respectively [21]. Despite the fact that the majority of individuals over the age of 65 years were enrolled in the program, this age group demonstrated the lowest rates of follow-up and adherence, although the rates were relatively high in Beijing and Shanghai [22]. Conversely, individuals aged between 18 and 44 years, who have the lowest enrollment rate, showed the highest rates of follow-up and adherence. It is not similar to common research results in which the oldest patients are the most adherent [19,20]. This phenomenon may be related to the fact that older individuals often have more complex health conditions, which CHCs cannot address well at present, leading them to generally prefer seeking treatment at higher-level medical institutions [23,24]. The observed low initial engagement of younger individuals with primary health care facilities may be attributed to a general mistrust or lower acceptance of these services among this demographic; the result is similar to that of a study conducted in Hong Kong [25]. Despite the low proportion of young patients who initially seek care at primary health centers following a hypertension diagnosis, those who do engage exhibit the highest follow-up and regular follow-up rates across all age groups. This indicates a high level of acceptance and satisfaction with primary health care management among younger patients once they have accessed these services.

Regarding marital status, the enrollment rate among widowed and divorced individuals was the highest, exceeding that of single individuals by more than 3 times. It is possible that this group, having experienced significant life events, became more concerned about their health. However, without a partner to provide encouragement and support [26], they may find it difficult to maintain consistent health behaviors and this group had higher mortality [27]. As a result, despite their high enrollment rates, their adherence tended to be relatively low. Interestingly, however, single individuals had the highest adherence rate. Perhaps this is because single individuals who enrolled in the HMP tend to pay more attention to their own health and are more likely to take action to keep healthy, leading to higher adherence; this is similar to a finding in the United States that never-married individuals reported better health habits and had higher rates of protective factors compared to married or divorced/separated individuals [28]. Previous studies showed that, in general, blue-collar employees appeared less likely to participate in health promotion programs compared to white-collar workers [29], and they may not have enough time to participate in the program [30,31]. Conversely, in our research, industrial workers stood out with the highest rates of enrollment, follow-up, and adherence across all categories; this may be related to the fact that in Shenzhen, many primary health care centers operate until 9 PM, providing this group with sufficient time to access the programs. Furthermore, the data indicated that, regardless of age, individuals with lower educational levels tended to have higher rates for all 3 metrics. Previous research has also found that individuals with higher education levels may be less likely to participate in cancer screening or achieve favorable screening outcomes [32], suggesting the impact of education on health behaviors is complex.

Importantly, internal migrants demonstrated a significantly higher participation in the HMP, especially in terms of project inclusion and regular follow-up. The participation rate of internal migrants was more than 10% higher than that of local residents. This result is inconsistent with previous global and national studies on the health service utilization of internal migrants. Previous research has indicated that internal migrants face long-standing disadvantages due to various institutional, social, cultural, and other exclusionary factors [33]. These disadvantages often extend to health and health care access, limiting their access to health resources [34,35]. A review focusing on migrants’ and refugees’ health status and health care in Europe highlighted the issue of underuse of primary health care services among these populations [36]. A study [37] based on the 2017 National Migrants Dynamic Monitoring Special Survey in China revealed that, compared to the registered population, the floating population is at a disadvantage in utilizing basic public health services. Most studies [38,39] focusing on hypertension management in China showed that the rate of hypertension management at primary health care centers among the floating population was less than 30% and as low as 2.3% in Hunan province [40], but our study showed that this situation does not exist in Shenzhen. In fact, among the hypertensive population in Shenzhen, on the contrary, internal migrants are better able to utilize primary health care services than locals. The Shenzhen municipal government has invested significantly in ensuring that all residents can access health care services within a 15-minute radius, making it highly convenient for the entire population. Additionally, the city provides free blood pressure screening at CHCs for all individuals aged ≥18 years, which promotes accessibility and equity in hypertension management services [41]. Certain districts in Shenzhen supply free medication to hypertensive patients enrolled in the program [42]. For most internal migrants, free health care services hold strong appeal due to their economic constraints. Some internal migrants came to Shenzhen to help their son/daughter look after children [43], so they or their son/daughter may care more about their health and they usually have enough time to go to a CHC. Insurance is considered an important factor for the utilization of health services for internal migrants and immediate reimbursement of medical insurance can significantly increase inpatient utilization, promoting health [44]. The statistical model showed that insurance type is an important factor influencing the 3 hypertension management rates studied. However, there were notable differences between local residents and internal migrants. For local residents, those with urban resident insurance were more likely to participate in the HMP compared to those with urban employee insurance. In contrast, for internal migrants, those with other insurance types were less likely to participate compared to those with urban employee insurance. It takes less money to get treatment and medicine in CHCs compared with upper health care facilities, especially for people who do not have native health insurance or cannot get immediate reimbursement.

Our analysis revealed several intriguing trends regarding HMP engagement among different demographic groups in Shenzhen. Previous studies conducted in Beijing and Shanghai have also found variations in hypertension management across different demographic factors, including age, gender, education level, workload, marital status, residential area, and annual household income [45,46]. Among individuals aged 18‐44 years, the enrolled rate of internal migrants was nearly 18% higher than that of local residents. This discrepancy may be attributed to the comparatively better economic conditions of local residents, who do not face the housing issues often encountered by migrants [47,48]. They may just go to upper hospitals when they think necessary, while internal migrants are more likely to utilize CHCs as the first choice for their routine health problems [49,50]. Additionally, the presence of their parents may lead locals to prioritize their own health less than migrants, who often lack familial support [51] and thus might be more vigilant about their health. Interestingly, in the divorced or widowed demographic, the follow-up rate among local residents surpassed that of internal migrants. Moreover, among local residents, the follow-up rate for this group was higher than that of married individuals. This finding contrasts with conventional research, which typically suggests that marriage promotes greater health awareness and engagement with health care services while the effects of bereavement usually lead to less health care utilization [52]. For married migrant individuals, having a spouse provides them with companionship [53] and significant support from their partner, whereas local residents can receive support from other relatives. This may cause internal migrants, compared to local residents, to have a more limited support system, making the protective effects of marriage on their health more pronounced. In contrast, local residents who are divorced or widowed may have access to a broader range of social support. The adherence rate showed an increasing divergence between local residents and internal migrants as age groups progress. Internal migrants tended to exhibit greater concern for their health after relocating to a new environment, which may explain this trend. Despite the fact that, among those with an undergraduate education or higher, the enrolled rate and follow-up rate for local residents were higher than those for internal migrants, the adherence rate for local residents remained lower than that of internal migrants. This suggests that, while highly educated local residents may initially engage with health care services, they may not maintain the same level of adherence as their migrant counterparts.

Some research showed that there exists vast disparities in health insurance coverage and health service utilization between floating populations and local residents; most of the floating population has no timely access to primary or some other kinds of health care service [39], which cause the poor control of chronic diseases. In our study, it appears that health insurance played a less significant role in the disparity of participation in the HMP between local residents and internal migrants.

From a district and community level, hypertensive patients were experiencing health inequity in hypertension treatment and prevention. The potential reasons included population, economic reasons, and policy differences. The populations of Longgang, Longhua, and Nanshan in Shenzhen are large and concentrated; in addition, the health care services are mature and systematic. Hence, the HMP is better than in other regions such as Yantian and Pingshan districts. Similarly, the wealthy regions had higher enrolled, follow-up, and adherence rates compared to the relatively poor regions, namely Yantian and Pingshan districts. These finding were consistent with previous research. A large national survey during 2004‐2018 found a provincial difference in awareness, treatment, and control is partly correlated with per capita gross domestic product [54]. Richer provinces are more successful in controlling hypertension. Globally, the proportions of hypertension awareness, treatment, and blood pressure control are low [55], particularly in low- and middle-income countries, and few comprehensive assessments of the economic impact of hypertension exist [56,57]. In terms of policy differences, the hypertensive patients who participate in the HMPs of CHCs could get free prescription medicines, blood pressure measurement services, and blood glucose measurement services once per quarter. The policy is different in different districts in Shenzhen, which may have caused the observed difference in enrolled, follow-up, and adherence rate. Furthermore, when it comes to health equity between internal migrants and native citizens in Shenzhen, the difference in enrolled, follow-up, and adherence rates within internal migrants was higher than that within local residents. This may be caused by different health care service supply situations toward internal migrants within different districts and communities [58]. The same problems were found in Ireland, Portugal, Spain, and the United States [59].

### Limitations and Strengths

This study represents a pioneering effort to examine the participation of internal migrants and local residents in HMPs, thereby highlighting the equity of internal migrants’ utilization of primary health care services under current Chinese hypertension management policies. By focusing on the specific involvement of diagnosed hypertension patients in HMPs, this research offers valuable insights into the accessibility and fairness of these services for internal migrants. The data utilized in this study were derived entirely from the primary health care system, ensuring its authenticity and reliability. However, this study has several limitations. It exclusively addressed the participation of hypertension patients in primary HMPs, which means that it did not account for patients who may receive regular treatment at other medical institutions without ever visiting CHCs. Consequently, the findings may not fully represent the hypertension management engagement of all patients citywide. Furthermore, the study was centered on participation rates within the HMP and did not capture the ultimate blood pressure control outcomes of the subjects. As a result, it is not possible to ascertain the differences in blood pressure control outcomes that may arise from varying levels of management participation.

Future research should consider integrating data from multiple health care providers and tracking long-term health outcomes to provide a more comprehensive understanding of hypertension management across diverse patient populations.

### Conclusions

Based on our research, of the 1,160,214 hypertensive patients, 642,250 were enrolled in the HMP, 48,848 were included in the followed up population, and 34,186 were adherents. Notably, internal migrants demonstrated a significantly higher participation in the HMP. Furthermore, populations with different demographic and socioeconomic characteristics and in different regions showed different rates. Given these findings, there is the potential to enhance the outreach and engagement of native, younger, and single populations through targeted promotional strategies.

## Supplementary material

10.2196/65548 Multimedia Appendix 1 Two-proportion *z*-test for enrollment rates, follow-up rates, and adherence rates between local residents and internal migrants.

10.2196/65548 Multimedia Appendix 2 Multivariable logistic regression analysis of the enrollment, follow up, and adherence of different groups in Shenzhen.

## Abbreviations

CHC: Community Health Center
HMP: hypertension management program
